# NANOG Amplifies STAT3 Activation and They Synergistically Induce the Naive Pluripotent Program

**DOI:** 10.1016/j.cub.2013.12.040

**Published:** 2014-02-03

**Authors:** Hannah T. Stuart, Anouk L. van Oosten, Aliaksandra Radzisheuskaya, Graziano Martello, Anzy Miller, Sabine Dietmann, Jennifer Nichols, José C.R. Silva

**Affiliations:** 1 Wellcome Trust-Medical Research Council Cambridge Stem Cell Institute, University of Cambridge, Tennis Court Road, Cambridge CB2 1QR, UK; 2 Department of Biochemistry, University of Cambridge, Tennis Court Road, Cambridge CB2 1GA, UK; 3 Department of Physiology, Development and Neuroscience, University of Cambridge, Downing Street, Cambridge CB2 3EG, UK

## Abstract

Reprogramming of a differentiated cell back to a naive pluripotent identity is thought to occur by several independent mechanisms. Two such mechanisms include NANOG and activated STAT3 (pSTAT3), known master regulators of naive pluripotency acquisition [1, 2, 3, 4, 5]. Here, we investigated the relationship between NANOG and pSTAT3 during the establishment and maintenance of naive pluripotency. Surprisingly, we found that NANOG enhances LIF signal transduction, resulting in elevated pSTAT3. This is mediated, at least in part, by suppression of the expression of the LIF/STAT3 negative regulator SOCS3. We also discovered NANOG to be limiting for the expression of KLF4, a canonical “Yamanaka” reprogramming factor [6] and key pSTAT3 target [2, 7, 8]. KLF4 expression resulted from the codependent and synergistic action of NANOG and pSTAT3 in embryonic stem cells and during initiation of reprogramming. Additionally, within 48 hr, the combined actions of NANOG and pSTAT3 in a reprogramming context resulted in reactivation of genes associated with naive pluripotency. Importantly, we show that NANOG can be bypassed during reprogramming by exogenous provision of its downstream effectors, namely pSTAT3 elevation and KLF4 expression. In conclusion, we propose that mechanisms of reprogramming are linked, rather than independent, and are centered on a small number of genes, including NANOG.

## Highlights

• NANOG amplifies STAT3 activation
• NANOG and STAT3 synergistically induce KLF4 expression
• NANOG and STAT3 rapidly induce naive gene expression in a reprogramming context
• Combined STAT3 and KLF4 bypass NANOG in reprogramming

## Teaser

Stuart et al. connect NANOG with STAT3 activation, two key factors for naive pluripotency establishment. They identify KLF4 expression as lying downstream of a synergistic relationship between NANOG and pSTAT3. Functionally, STAT3 activation and KLF4 expression bypass the requirement for NANOG in reprogramming.

## Results

### NANOG Amplifies STAT3 Activation

We first investigated the effect of NANOG on STAT3 activation in embryonic stem cells (ESCs), since the pluripotency network is established and functional in this cellular context. Although NANOG and STAT3 are essential for embryonic naive pluripotency establishment [4, 9], they promote but are not required for in vitro ESC maintenance [10, 11, 12, 13, 14]. This permits the study of *Nanog*^−/−^ and *Stat3*^−/−^ ESCs and suggests differences between network requirements for naive pluripotency establishment versus maintenance.

**Figure 1:**
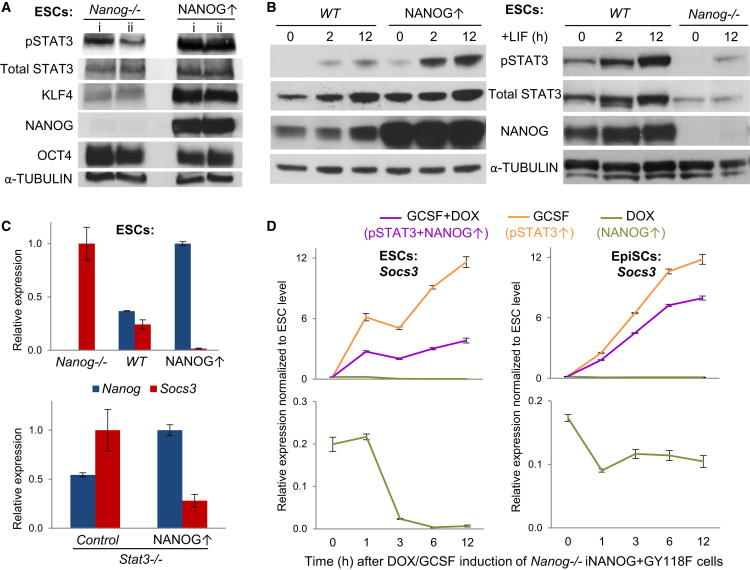
NANOG Amplifies STAT3 Activation (A) Western blot analysis of pSTAT3, total STAT3, and KLF4 protein expression in *Nanog*^−/−^ and constitutively NANOG-overexpressing ESCs cultured in steady-state serum+LIF conditions and harvested on two separate days (i and ii). OCT4 expression confirmed undifferentiated status. (B) Western blot analysis of pSTAT3 and total STAT3 protein expression in wild-type, constitutively NANOG-overexpressing, and *Nanog*^−/−^ ESCs in response to LIF stimulation for 0, 2, and 12 hr. Positive feedback of pSTAT3 induction on STAT3 and NANOG expression was observed, in accordance with previous work [2, 7, 15]. Selection for pluripotent cells was maintained throughout. Left: LIF was withdrawn from serum+LIF culture for 36 hr prior to readdition. Right: ESCs were cultured in 2i for at least 7 days prior to LIF addition. Different conditions were used to provide the most informative comparisons, since 2i boosts the expression of endogenous NANOG compared to serum conditions [16]. (C and D) qRT-PCR analysis of gene expression, relative to *Gapdh* and normalized either to the highest value (C) or to serum+LIF ESC level (D). Data shown are the mean of three technical replicates and are from one of two representative experiments. Error bars indicate ±SD. (C) *Nanog* and *Socs3* expression in ESCs cultured in 2i without LIF for at least 7 days. Top: *Nanog*^−/−^, wild-type, and constitutively NANOG-overexpressing ESCs. Bottom: control and NANOG-overexpressing *Stat3*^−/−^ ESCs. (D) Top left: *Socs3* expression in *Nanog*^−/−^ iNANOG+GY118F ESCs after induction with dox and/or GCSF in 2i. Top right: *Socs3* expression in *Nanog*^−/−^ iNANOG+GY118F EpiSCs after induction with dox and/or GCSF in FGF2+ActivinA. Bottom: zoomed-in view of the respective dox inductions. See also Figure S1.

Active pSTAT3 lies downstream of a LIF-stimulated tyrosine kinase signaling cascade [1] (Figure S1A available online). Wild-type, *Nanog*^−/−^, and constitutively NANOG-overexpressing ESCs were harvested from steady-state and LIF-induction cultures. Western blotting revealed that higher NANOG both increased steady-state pSTAT3 levels (Figures 1A and S1B) and enhanced ESC sensitivity to LIF stimulation (Figure 1B).

To explain how NANOG drives pSTAT3 elevation without directly binding the STAT3 protein or gene [17, 18], we examined the effect of NANOG on components of the LIF/STAT3 signaling pathway (Figure S1A). By quantitative RT-PCR (qRT-PCR), no correlation was reliably found between NANOG and transcript levels of the positive signal transducers *Lif*, *Lifr*, *Gp130*, *Jak2*, and *Stat3* (data not shown), despite NANOG binding to *Lif*, *Lifr*, and *Gp130* gene regulatory sequences in ESCs according to published chromatin immunoprecipitation sequencing data [18]. However, NANOG also binds the *Socs3* gene (Figure S1C), which is a negative regulator of STAT3 activation [19, 20]. This prompted our hypothesis that NANOG represses *Socs3* transcription.

Since *Socs3* transcription is upregulated by pSTAT3 to form a classic negative feedback loop [20, 21], determination of whether NANOG causes *Socs3* repression is obfuscated by the effect of pSTAT3 on *Socs3.* To disentangle the opposing yet interconnected influences of NANOG and pSTAT3 on *Socs3* expression, we designed experiments in which the pSTAT3 level is not significantly influenced by NANOG. ESCs can be maintained without exogenous LIF by 2i medium [10], which contains small molecules CHIR99021 (chiron) and PD0325901 (PD03) that inhibit GSK3 and MEK, respectively. In the absence of LIF-stimulated *Socs3* activation, the effect of NANOG on *Socs3* transcription can be assessed. From qRT-PCR analysis of wild-type, *Nanog*^−/−^, and constitutively NANOG-overexpressing ESCs cultured in 2i, a strong negative correlation was evident between *Nanog* and *Socs3* expression levels (Figure 1C). Furthermore, in the absolute absence of pSTAT3 in *Stat3*^−/−^ ESCs, those constitutively overexpressing NANOG exhibited a lower *Socs3* level (Figure 1C).

STAT3 can be specifically activated by GCSF stimulation of the GY118F receptor transgene [22, 23, 24] (Figure S1A). When GCSF/GY118F are used in the absence of LIF, *Socs3* downregulation by NANOG should have little or no effect on pSTAT3 levels; GY118F is insensitive to SOCS3 repression, and nearly all STAT3 activation will be attributable to GY118F rather than LIFR-GP130. In addition, we generated a doxycycline (dox)-inducible *Nanog* transgene (iNANOG) (Figure S1D). Dox induction of NANOG expression rapidly led to enriched NANOG binding at the *Socs3* gene in ESCs, consistent with direct transcriptional regulation (Figure S1C). In *Nanog*^−/−^ ESCs containing both iNANOG and GY118F, NANOG expression and STAT3 activation were induced separately and in combination in 2i. Induction of NANOG alone caused *Socs3* repression, while *Socs3* induction in response to STAT3 activation was reduced by 60% when NANOG was also induced (Figure 1D). This suggests that *Socs3* repression is a mechanism by which NANOG augments LIF signal transduction in ESCs, resulting in higher levels of active pSTAT3. The same trends were observed in *Nanog*^−/−^ iNANOG+GY118F postimplantation epiblast stem cells (EpiSCs) in standard FGF2+ActivinA conditions (Figure 1D), demonstrating that NANOG-mediated *Socs3* repression is not restricted to ESCs and may be of functional relevance during NANOG-driven reprogramming.

### NANOG and pSTAT3 Synergistically Upregulate KLF4

**Figure 2:**
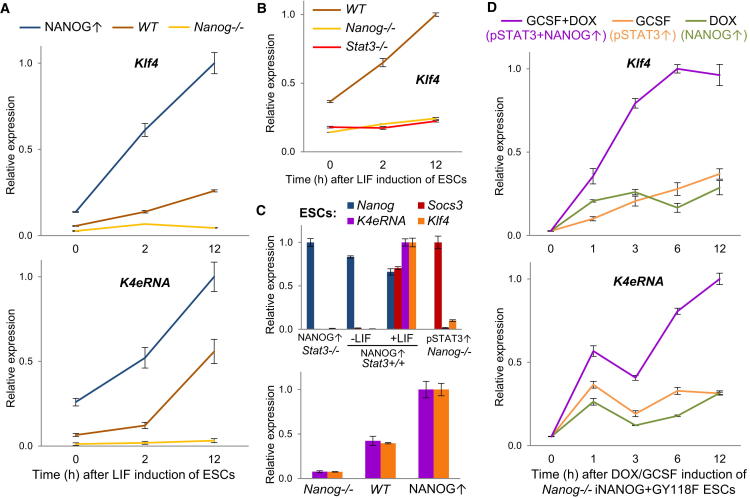
NANOG and pSTAT3 Synergistically Upregulate KLF4 qRT-PCR analysis of gene expression relative to *Gapdh* and normalized to the highest value. Data shown are the mean of three technical replicates and are from one of two representative experiments. Error bars indicate ±SD. (A) *Klf4* and *K4eRNA* expression in *Nanog*^−/−^, wild-type, and constitutively NANOG-overexpressing ESCs in response to LIF stimulation. LIF was withdrawn from serum culture for 36 hr prior to readdition. Selection for pluripotent cells was maintained throughout. (B) *Klf4* expression in *Stat3*^−/−^, *Nanog*^−/−^, and wild-type ESCs in response to LIF stimulation. LIF was added after at least 7 days in 2i without LIF. (C) Top: *Klf4* and *K4eRNA* expression in NANOG-overexpressing *Stat3*^−/−^, NANOG-overexpressing *Stat3*^*+/+*^, and STAT3-hyperactivated (GCSF/GY118F) *Nanog*^−/−^ ESCs in basic conditions with or without LIF. *Socs3* expression indicates STAT3 activation as appropriate. Bottom: *Klf4* and *K4eRNA* expression in *Nanog*^−/−^, wild-type, and constitutively NANOG-overexpressing ESCs cultured in steady-state serum+LIF. (D) *Klf4* and *K4eRNA* expression in *Nanog*^−/−^ iNANOG+GY118F ESCs after induction with dox and/or GCSF in 2i. See also Figure S2.

To further explore our newfound mechanistic link between NANOG and LIF/STAT3 signaling, we investigated the effect of NANOG on expression of LIF/STAT3 targets in ESCs. In the steady-state presence of LIF, we found strong positive correlation between levels of NANOG and KLF4, a canonical pSTAT3 target [2, 7, 8] (Figures 1A and S1B). Interestingly, in response to LIF stimulation, *Klf4* upregulation required NANOG to be present, while NANOG overexpression cooperated with LIF to substantially increase the rate and levels of *Klf4* induction (Figures 2A and 2B). In agreement with previous work [8], modest upregulation of NANOG was found in response to LIF (Figures 1B and S2A).

The correlation between NANOG and *Klf4* was abrogated in the absence of pSTAT3 in *Stat3*^*+/+*^ ESCs without LIF and in *Stat3*^−/−^ ESCs (Figure 2C), showing that NANOG-driven *Klf4* upregulation was pSTAT3 dependent. However, the effect of NANOG on *Klf4* transcription was not solely attributable to pSTAT3 elevation, since STAT3 hyperactivation in the absence of NANOG could not rescue *Klf4* expression (Figure 2C).

The relationship between NANOG, pSTAT3, and *Klf4* expression was further dissected using *Nanog*^−/−^ iNANOG+GY118F ESCs in 2i without LIF. Together, induction of NANOG expression and STAT3 activation elicited *Klf4* upregulation in a synergistic manner compared to induction of either factor alone (Figure 2D). This synergistic action of NANOG and pSTAT3 is specific to *Klf4*: other pluripotency factors did not respond in this striking manner, including NANOG-target *Esrrb* [25] and pSTAT3-target *Klf5* [7, 8] (Figure S2B).

Since NANOG and pSTAT3 both bind the *Klf4* enhancer (Figure S2C), it is likely that they regulate *Klf4* transcription directly. The *Klf4* enhancer lies around 60 kb downstream of the *Klf4* transcription start site and was recently identified as an archetypal “super-enhancer” [26]. We found novel noncoding RNA to be expressed from the *Klf4* enhancer in ESCs, and we termed it *K4eRNA* (Figure S2C). Given that *K4eRNA* expression positively correlates with *Klf4*, responding to NANOG and LIF/STAT3 in the same synergistic manner (Figures 2A, 2C, and 2D), we hypothesize that *K4eRNA* is a *cis* activator of *Klf4* transcription.

### NANOG and pSTAT3 Induce Rapid and Efficient Reactivation of Naive Genes

EpiSC reprogramming requires reversion from primed to naive pluripotency and thus provides an excellent system in which to study naive pluripotency acquisition. Conversion of EpiSCs to iPSCs does not occur simply in naive-state culture conditions, but can be driven by a minimum of one factor [27]. It is known that NANOG overexpression and STAT3 hyperactivation together increase EpiSC reprogramming efficiency in a synergistic manner [1]. Since we have mechanistically linked NANOG and pSTAT3 in ESCs where the pluripotency network is fully operational, we turned to EpiSCs to study the role of these connected mechanisms during naive pluripotency establishment.

**Figure 3:**
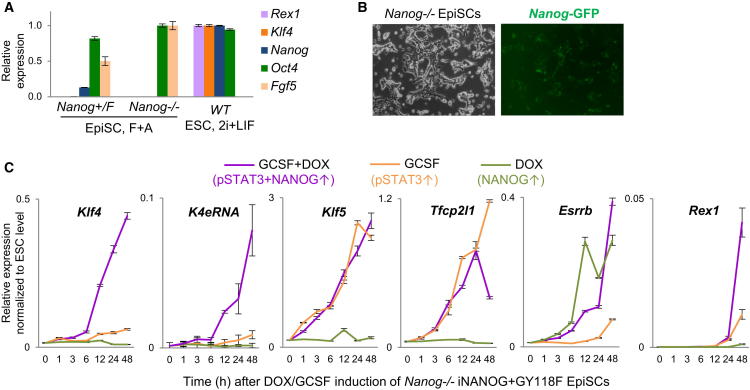
NANOG and pSTAT3 Induce Rapid and Efficient Reactivation of Naive Genes (A) qRT-PCR analysis of *Nanog*^−/−^ and *Nanog*^+/Flox^ (*Nanog*^+/F^) EpiSCs derived from *Nanog*^−/F^ and *Nanog*^+/F^ littermate embryos, cultured in FGF2+ActivinA (F+A), compared to wild-type ESCs in 2i+LIF. Lack of *Nanog* expression in *Nanog*^−/−^ EpiSCs confirmed the null genotype, while their expression of *Oct4* and *Fgf5* but not *Rex1* or *Klf4* confirmed their EpiSC identity. Gene expression was measured relative to *Gapdh* and normalized to the highest value. Data shown are the mean of three technical replicates and are from one of two representative experiments. Error bars indicate ±SD. (B) Representative phase and *Nanog-*GFP images of the *Nanog*^−/−^ EpiSCs derived from *Nanog*^−/F^ embryos, in FGF2+ActivinA conditions. GFP reporter is under the endogenous *Nanog* promoter of the floxed null allele (Figure S3A). Images are 1122 μm by 839 μm. (C) qRT-PCR analysis of *Klf4*, *K4eRNA*, *Klf5*, *Tfcp2l1*, *Esrrb*, and *Rex1* expression in *Nanog*^−/−^ iNANOG+GY118F EpiSCs after induction with dox and/or GCSF in FGF2+ActivinA. Gene expression was measured relative to *Gapdh* and normalized to serum+LIF ESC level = 1. Data shown are the mean of three technical replicates and are from one of two representative experiments. Error bars indicate ±SD. See also Figure S3.

The iNANOG+GY118F system provides a powerful platform for the quantitative dissection of NANOG and pSTAT3 mechanisms, as they can be induced separately and in combination within a single cell line. We generated *Nanog*^−/−^ background EpiSCs (Figure 3A, 3B, and S3A–S3C) to eliminate confounding endogenous *Nanog* expression, and maintained EpiSC FGF2+ActivinA culture conditions so that putative reprogramming kinetics could be ascribed exclusively to transgene induction. Strikingly, NANOG and pSTAT3 codependently reactivated *Klf4* and *K4eRNA* in this distinct cellular and environmental context (Figure 3C). This demonstrates that their effect on *Klf4* is not an ESC-specific phenomenon and may be of functional relevance for NANOG/pSTAT3-driven reprogramming. We observed activation of naive pluripotency marker *Rex1* at 48 hr (Figure 3C). Since *Rex1* did not respond to NANOG/pSTAT3 in this manner in ESCs (Figure S2B), we believe *Rex1* induction in EpiSCs to be indirect, indicating identity changes toward iPSCs within the population. Interestingly, *Rex1* activation positively correlates with *Klf4* expression.

Known NANOG-target *Esrrb* [25] responded to dox induction of NANOG expression in EpiSCs (Figure 3C). Similarly, known pSTAT3 targets *Klf5* [7, 8] and *Tfcp2l1* [15, 28] were upregulated after GCSF induction of STAT3 activation (Figure 3C). However, the synergistic response of *Klf4* to NANOG and pSTAT3 remains unique. In total, NANOG and pSTAT3 rapidly reactivated many key components of the naive pluripotency network to near ESC level, shedding light on their ability to drive fast and efficient reprogramming. This is even more remarkable when taking into account that the assay used EpiSC culture conditions instead of conditions promoting reprogramming or ESC self-renewal.

### Combined pSTAT3 and KLF4 Bypass NANOG in Reprogramming

Although NANOG is dispensable for pluripotency maintenance [11], it is required for establishment of the pluripotent epiblast during preimplantation embryonic development [4]. Correspondingly, NANOG is essential for naive pluripotency establishment during conventional in vitro reprogramming experiments [4]. Rescue of *Nanog*^−/−^ reprogramming thus provides a means of functionally testing proposed downstream mechanisms of NANOG.

**Figure 4:**
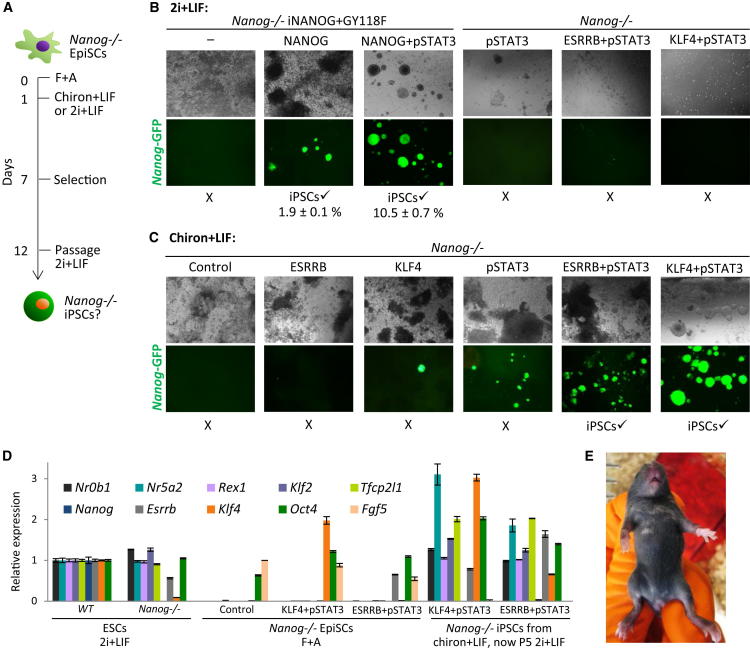
Combined pSTAT3 and KLF4 Bypass NANOG in Reprogramming (A) Schematic depicting the protocol for reprogramming of *Nanog*^−/−^ EpiSCs containing *βgeo* and *GFP* reporters under the endogenous *Nanog* promoters (Figure S3A). EpiSCs were plated in FGF2+ActivinA (F+A) and, after 1 day, medium was switched to chiron+LIF or 2i+LIF to prompt reprogramming. On day 7, G418, FGFR inhibitor, and ALK inhibitor were added to select for emergent iPSCs. On day 12, iPSCs were passaged into 2i+LIF. (B) Representative phase and *Nanog-*GFP images taken on day 12 of reprogramming in 2i+LIF. The mean number of GFP^+^ iPSC colonies is indicated as the percentage of cells initially plated, ±SD (n = 3 biological replicates). *Nanog*^−/−^ iNANOG+GY118F EpiSCs generated GFP^+^ iPSCs in 2i+LIF only if dox or dox+GCSF were supplied during reprogramming, to induce NANOG expression and STAT3 activation, respectively. *Nanog*^−/−^ EpiSCs with activated STAT3 (GCSF/GY118F) and constitutive ESRRB or KLF4 expression did not generate GFP^+^ iPSCs in 2i+LIF. (C) Representative phase and *Nanog-*GFP images taken on day 12 of reprogramming in chiron+LIF. Quantification of reprogramming efficiency by GFP^+^ colony counting was inappropriate in this case, since chiron+LIF is a permissive medium compared to 2i+LIF and yielded some GFP^+^ colonies that were not true iPSCs. After passaging in 2i+LIF, clean GFP^+^ iPSC lines were obtained as indicated. (D) qRT-PCR analysis of gene expression in chiron+LIF-derived *Nanog*^−/−^ iPSCs after passaging in 2i+LIF, compared to parental EpiSC lines in FGF2+ActivinA (F+A) and wild-type and *Nanog*^−/−^ ESCs in 2i+LIF. Expression patterns of *Nanog*, *Esrrb*, and *Klf4* verified the genotypes. Reprogramming to naive iPSCs was confirmed by reactivation of naive genes, maintenance of *Oct4*, and repression of *Fgf5* expression. Gene expression was measured relative to *Gapdh* and normalized to wild-type ESC level, except *Fgf5*, which was normalized to control EpiSCs. Data shown are the mean of three technical replicates and are from one of two representative experiments. Error bars indicate ±SD. (E) Chimeric mouse obtained after injection of pSTAT3+KLF4 EpiSC-derived *Nanog*^−/−^ iPSCs into C57/BL6 blastocysts. Agouti coat color indicates chimeric contribution (MF1/129 background). See also Figure S4.

We have described two new NANOG mechanisms: pSTAT3 elevation by SOCS3 repression and KLF4 upregulation in cooperation with pSTAT3. Therefore, we investigated the ability of pSTAT3 and KLF4 to rescue reprogramming of *Nanog*^−/−^ EpiSCs. We also tested ESRRB, since it has previously been reported as a NANOG downstream target able to bypass NANOG in reprogramming of ESC-derived EpiSCs [25]. Expression of *GFP* and *βgeo* under the control of endogenous *Nanog* promoters provide visual and selective reporters in our system, since EpiSCs cannot survive long-term in the reprogramming culture conditions (Figure 4A).

Forced expression of NANOG, ESRRB, KLF4 or pSTAT3 individually can drive reprogramming of *Nanog*^*+/+*^ EpiSCs in 2i+LIF conditions [1, 4, 25, 27]. We verified the ability of our *Nanog*^−/−^ EpiSCs to generate iPSCs in 2i+LIF when rescued by NANOG expression and confirmed that STAT3 activation in conjunction with NANOG expression drives rapid reprogramming at high efficiency (Figure 4B). However, in the absence of NANOG, individual and combined overexpression of ESRRB, KLF4, and pSTAT3 could not rescue reprogramming in 2i+LIF (Figure 4B).

In parallel, we tried to rescue *Nanog*^*−/−*^ reprogramming with individual and combined overexpression of ESRRB, KLF4, and pSTAT3 in chiron+LIF (Figure 4C) and in serum+LIF (data not shown). To our surprise, we found that ESRRB overexpression was unable to drive *Nanog*^−/−^ EpiSC reprogramming in any condition. We speculate that this is due to differences in reprogramming propensity between our EpiSCs derived from *Nanog*^−/F^ embryos and ESC-derived secondary EpiSC systems [25]. Individually, KLF4 and pSTAT3 also failed to yield iPSCs. Although they initially generated GFP^+^ colonies in chiron+LIF (Figure 4C), these lacked the capacity to self-renew after passaging into 2i+LIF, suggesting that reprogramming was incomplete.

In combination, pSTAT3+ESRRB and pSTAT3+KLF4 yielded *Nanog*^−/−^ GFP^+^ iPSCs in chiron+LIF conditions (Figure 4C). Reprogramming occurred with the highest rate and efficiency with pSTAT3+KLF4. Although *Nanog*^−/−^ iPSCs could not be established in 2i+LIF, once naive pluripotency was established in chiron+LIF, *Nanog*^−/−^ iPSCs could be passaged indefinitely in 2i+LIF, consistent with known discrepancies between NANOG requirement in pluripotency establishment versus maintenance. After passaging in 2i+LIF, the gene expression profile and observed chimeric competence of *Nanog*^−/−^ iPSCs formally demonstrated their reacquisition of a naive pluripotent program (Figures 4D and 4E). This highlights pSTAT3 activation as a key functional mechanism acting downstream of NANOG, which, in conjunction with overexpression of either NANOG-target KLF4 or ESRRB, can efficiently rescue *Nanog*^−/−^ reprogramming. It is of interest to note that KLF4 but not NANOG overexpression can drive EpiSC reprogramming in the presence of JAK inhibitor [1], further supporting the placement of NANOG upstream and KLF4 downstream of STAT3 activation during reprogramming.

NANOG is dispensable for the initial formation of reprogramming intermediates (pre-iPSCs) from somatic cells, but is essential for pre-iPSCs to transit to naive pluripotency in 2i+LIF [4] (confirmed in Figures S4A and S4B). Since *Nanog*^−/−^ EpiSCs were able to reprogram in chiron+LIF but not 2i+LIF, we tested whether *Nanog*^−/−^ pre-iPSCs could also reprogram in alternative conditions. From these, we successfully obtained *Nanog*^−/−^ iPSCs in chiron+LIF and KSR+LIF conditions, albeit with low speed and efficiency (Figures S4C–S4E). Microarray analysis revealed that pre-iPSC-derived and EpiSC-derived *Nanog*^−/−^ iPSCs clustered closely with both wild-type and *Nanog*^−/−^ ESCs, demonstrating reprogramming to a naive pluripotent identity (Figure S4F). It should be noted that retroviral *Klf4* and exogenous LIF provided the reprogramming impetus for these pre-iPSCs, again implicating pSTAT3 and KLF4 in the rescue of *Nanog*^−/−^ reprogramming.

## Discussion

We connect NANOG with the activation of STAT3, two major mechanisms for the establishment and maintenance of naive pluripotency. Our finding that NANOG modulates signal transduction of extracellular cues adds a new dimension to the interplay between external environment and nuclear control networks to instate and reinforce cellular identity. We also provide mechanistic insight into the process of induced pluripotency by showing that expression of KLF4, a canonical “Yamanaka” factor, results from codependent and synergistic action between NANOG and pSTAT3. Interestingly, the only remaining factor to be used by all reprogramming protocols is LIF and consequently STAT3 activation, now linked to NANOG.

The role of NANOG is thus to build a naive pluripotent transcriptional network by concurrently inducing the expression of ESRRB, enhancing LIF/STAT3 signal transduction, and inducing KLF4 expression in cooperation with pSTAT3 (Figure S4G). Ultimately, combinations of these factors allow in vitro bypassing of NANOG for the establishment of a naive pluripotent cell state. However, the observed slower kinetics and reduced efficiency of *Nanog*^−/−^ somatic cell reprogramming imply the existence of further mechanisms by which NANOG operates. These may include additional downstream effectors of reprogramming and the activities of NANOG cofactors such as TET1/2 [29]. In this light, it is interesting to note that bypass of NANOG in reprogramming was enhanced by KSR medium (Figure S4C), which contains ascorbic acid, a powerful coactivator of dioxygenases such as the jumonji histone demethylases and TETs [30, 31, 32, 33, 34]. Additionally, it will be of future interest to ascertain why 2i conditions are detrimental to *Nanog*^−/−^ reprogramming.

Successful induction of naive pluripotency can be achieved by the combined actions of different culture environments with different sets of transgenes. This has led to the notion that iPSCs can be generated by different, independent reprogramming mechanisms acting in an additive, linear manner. In contrast to this, our newfound cooperative relationship between NANOG and STAT3 activity raises the possibility of an integrated reprogramming mechanism. Therefore, we propose that allegedly independent mechanisms of naive pluripotency induction may instead be linked and centered on a small group of genes including NANOG.

## Accession Numbers

The Gene Expression Omnibus accession number for the microarray data reported in this paper is GSE53529.

## Supplemental Information

Document S1. Supplemental Experimental Procedures and Figures S1–S4
