# Supplementary material for: NANOG Amplifies STAT3 Activation and They Synergistically Induce the Naive Pluripotent Program
Source: Curr Biol. 2014 Feb 3;24(3):340–6. doi: 10.1016/j.cub.2013.12.040 (PMC3982126; doi:10.1016/j.cub.2013.12.040)
Supplement: Document S1. Supplemental Experimental Procedures and Figures S1–S4 [file mmc1.pdf]

Current Biology, Volume *24*

Supplemental Information

NANOG Amplifies STAT3 Activation and They Synergistically Induce the Naive Pluripotent Program

Hannah T. Stuart, Anouk L. van Oosten, Aliaksandra Radzisheuskaya, Graziano Martello, Anzy Miller, Sabine Dietmann, Jennifer Nichols, and José C.R. Silva

Figure S1:

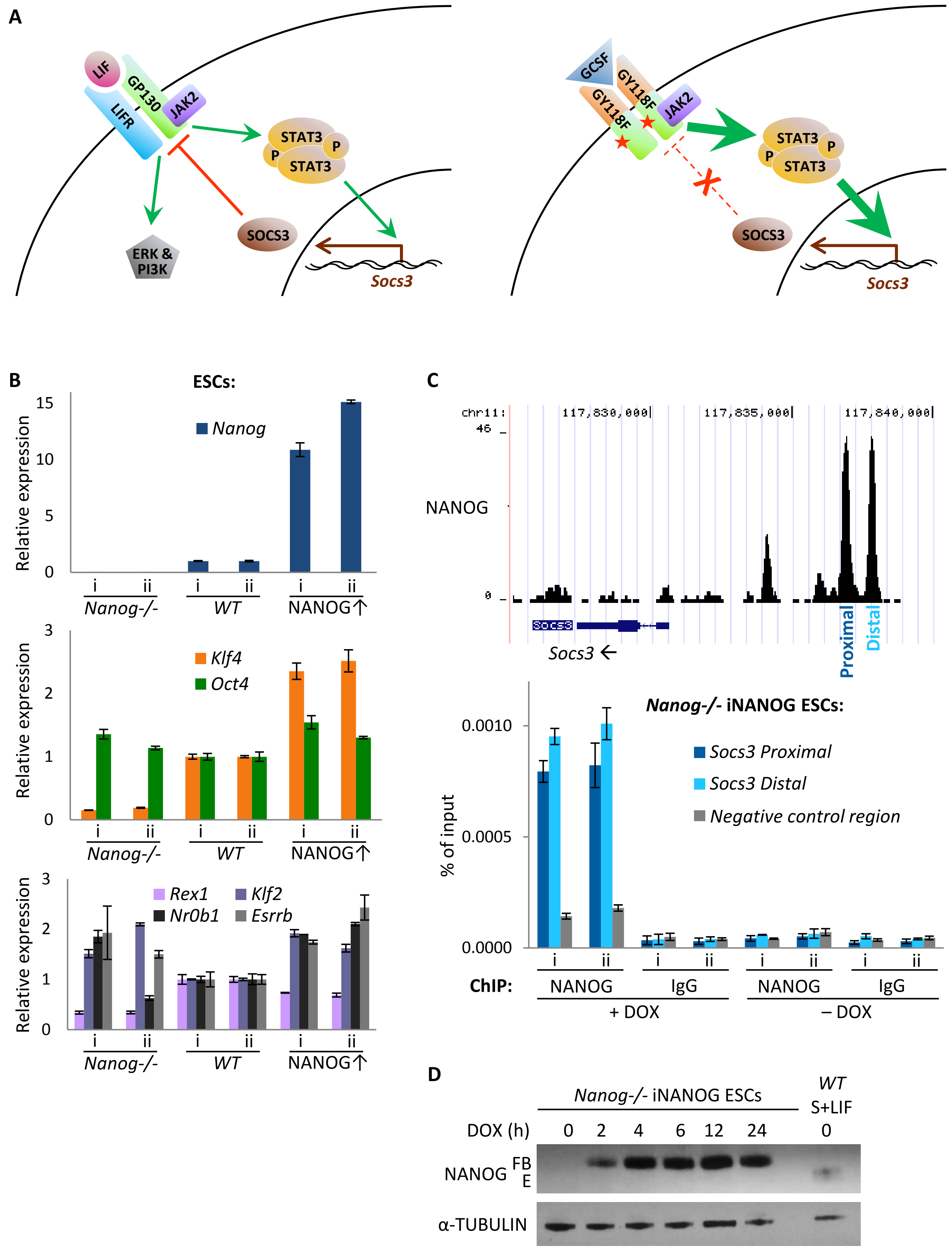
A
LIF
GP130
LIFR
JAK2
STAT3
P
ERK & PI3K
SOCS3
Socs3
GCSF
GY118F
B
ESCs:
Relative expression
Nanog
Nanog-/-
WT
NANOG↑
Klf4
Oct4
Rex1
Klf2
Nr0b1
Esrrb
C
chr11:
117,830,000
117,835,000
117,840,000
NANOG
Socs3 ←
Proximal
Distal
Nanog-/- iNANOG ESCs:
% of input
Socs3 Proximal
Socs3 Distal
Negative control region
ChIP:
NANOG
IgG
+ DOX
– DOX
D
Nanog-/- iNANOG ESCs
WT S+LIF
DOX (h)
0
2
4
6
12
24
NANOG
FBE
α-TUBULIN

Figure S1: NANOG amplifies STAT3 activation (Related to Figure 1)

(A) Simplified schematic comparing LIF/LIFR-GP130 and GCSF/GY118F signal transduction pathways. Left: LIF ligand binds the extracellular domains of LIFR and GP130, triggering phosphorylation and activation of JAK2 associated with the intracellular domain of GP130. JAK2 phosphorylates the intracellular domain of GP130, causing recruitment of latent cytoplasmic STAT3 to GP130 via the STAT3 SH2 phosphotyrosine-binding domain. JAK2 then phosphorylates tyrosine-705 of STAT3 (pSTAT3), allowing SH2-mediated homodimerization of pSTAT3, nuclear translocation and transcriptional activation of multiple target genes [S1-5]. *Socs3* transcription is rapidly and strongly induced by pSTAT3. SOCS3 binds to GP130-JAK2 complexes, prevents STAT3 binding to JAK2, and thus inhibits STAT3 phosphorylation and activation [S6, S7], forming a classic negative feedback loop in which pSTAT3 and SOCS3 levels oscillate in antiphase [S8]. Additionally, LIF signalling leads to activation of the MAPK/ERK and PI3-kinase pathways [S9, S10]. Right: Specific and sustained STAT3 activation can be achieved by supplying GCSF to cells expressing GY118F, a chimeric transmembrane receptor [S11-13]. The GY118F extracellular domain is that of the human GCSF receptor, whereas the transmembrane and intracellular domains are based on murine GP130, with a point mutation of tyrosine-118 to phenylalanine. The Y118F mutation prevents activation of MAPK/ERK and PI3-kinase pathways, and blocks SOCS3-mediated negative feedback by eliminating the SOCS3 binding site on GP130.

(B) RT-qPCR analysis of gene expression in *Nanog-/-*, wild-type and constitutively NANOG-overexpressing ESCs cultured in steady-state serum+LIF conditions and harvested on 2 separate days (i and ii). Expression was measured relative to *Gapdh* and normalized to the appropriate wild-type sample. *Oct4* expression confirmed undifferentiated status. *Rex1, Klf2,* and *Nr0b1* expression patterns confirmed that the strong positive correlation observed between *Nanog* and *Klf4* was specific. Data shown are the mean of 3 technical replicates. Error bars indicate ± s.d.

(C) Upper: Published ESC ChIP-seq data [S14] reveal that NANOG binds the upstream regulatory region of the *Socs3* gene. Arrow indicates the direction of *Socs3* transcription. Lower: ChIP analysis of NANOG binding at the regulatory region of *Socs3*. Proximal and distal NANOG binding sites are as indicated above. NANOG expression was dox-induced in *Nanog-/-* iNANOG ESCs in 2i culture and samples were harvested at t=0 and 3 hours. Data shown are the mean of 3 technical replicates from each of 2 experiments (i and ii). Error bars indicate ± s.d.

(D) Western blot analysis of NANOG protein expression in *Nanog-/-* iNANOG ESCs following dox induction in serum+LIF, compared to wild-type ESCs cultured in serum+LIF. FLAG- and biotin-tagged (FB) NANOG expressed from iNANOG was detected at a higher molecular weight than endogenous (E) NANOG.

Figure S2:

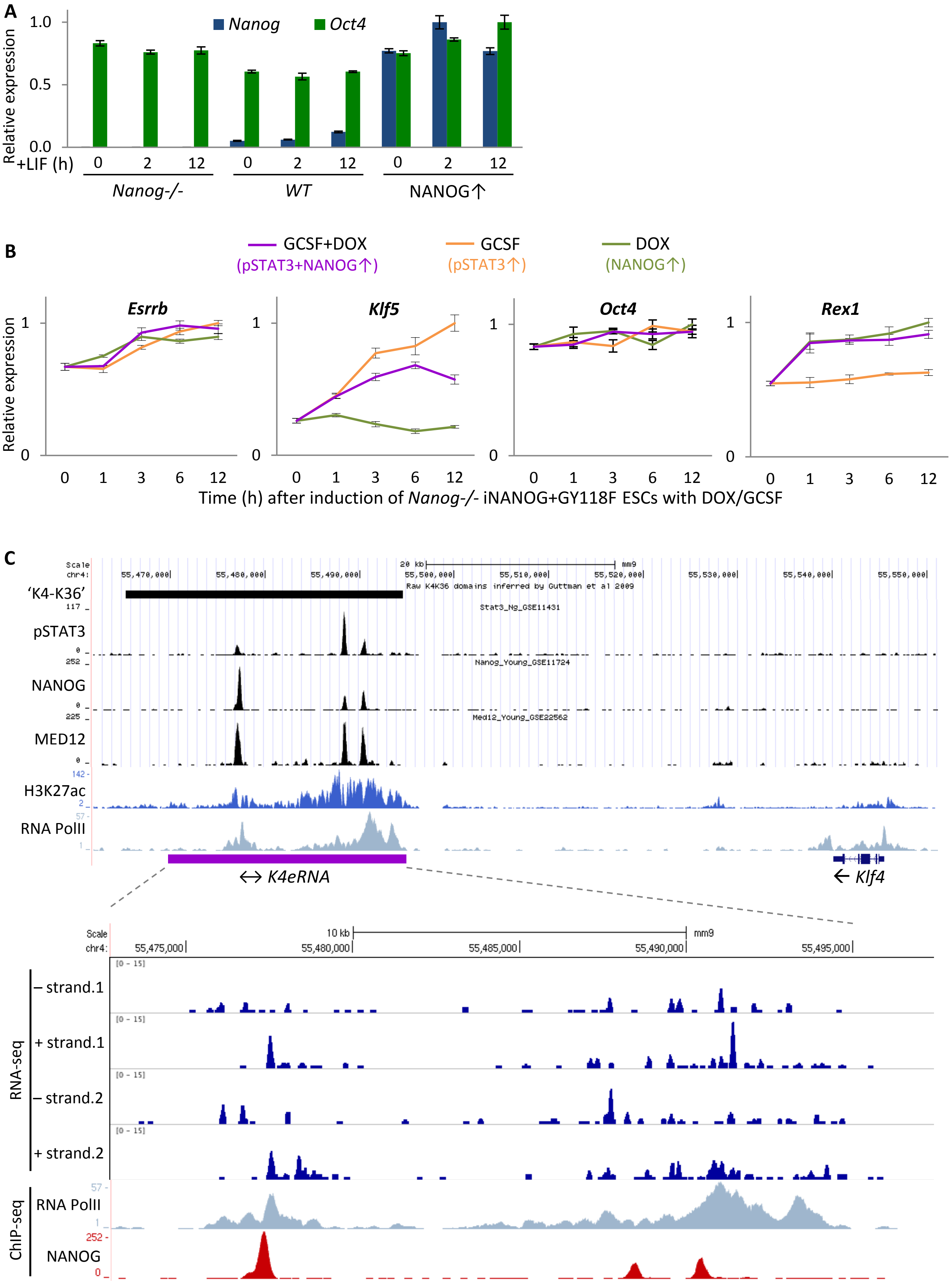
A
Nanog
Oct4
Relative expression
1.0
0.5
0.0
+LIF (h)
0
2
12
Nanog-/-
WT
NANOG↑
B
GCSF+DOX
(pSTAT3+NANOG↑)
GCSF
(pSTAT3↑)
DOX
(NANOG↑)
Esrrb
Klf5
Oct4
Rex1
Relative expression
Time (h) after induction of Nanog-/- iNANOG+GY118F ESCs with DOX/GCSF
C
'K4-K36'
pSTAT3
NANOG
MED12
H3K27ac
RNA PolII
↔ K4eRNA
← Klf4
Raw K4K36 domains inferred by Guttman et al 2009
Stat3_Ng_GSE11431
Nanog_Young_GSE11724
Med12_Young_GSE22562
– strand.1
+ strand.1
– strand.2
+ strand.2
RNA-seq
ChIP-seq
RNA PolII
NANOG

Figure S2: NANOG and pSTAT3 synergistically upregulate KLF4 (Related to Figure 2)

(A) RT-qPCR analysis of *Oct4* and *Nanog* expression, relative to *Gapdh* and normalized to the highest value. LIF was withdrawn from serum culture of *Nanog-/-*, wild-type and constitutively NANOG-overexpressing ESCs for 36 hours prior to re-addition. Selection for pluripotent cells was maintained throughout, and *Oct4* expression indicated undifferentiated status. Data shown are the mean of 3 technical replicates and are from 1 of 2 representative experiments. Error bars indicate ± s.d.

(B) RT-qPCR analysis of *Esrrb, Klf5, Oct4* and *Rex1* expression in *Nanog-/-* iNANOG+GY118F ESCs following induction with dox and/or GCSF in 2i. Gene expression was measured relative to *Gapdh* and normalized to the highest value. Data shown are the mean of 3 technical replicates and are from 1 of 2 representative experiments. Error bars indicate ± s.d.

(C) The *Klf4* gene locus shown on assembly mm9. Upper: Analysis of published ESC ChIP-seq data revealed pSTAT3, NANOG and Mediator (MED12) binding to the *Klf4* downstream enhancer. RNA PolII binding and H3K27ac enrichment suggest active transcription from this enhancer. Furthermore, a 'K4-K36 domain' is predicted here, which, in the absence of a known protein-coding gene, is thought to demarcate PolII-transcribed non-coding RNA [S15]. We found novel non-coding RNA to be expressed from the *Klf4* enhancer in ESCs, termed *K4eRNA,* the position of which is marked by the purple box. Arrows indicate the directions of *K4eRNA* and *Klf4* transcription. Lower: Published RNA-seq [S16] indicates bidirectional transcription from the *Klf4* downstream enhancer in wild-type ESCs in 2i+LIF, confirmed here by RT-PCR (data not shown).

Figure S3:

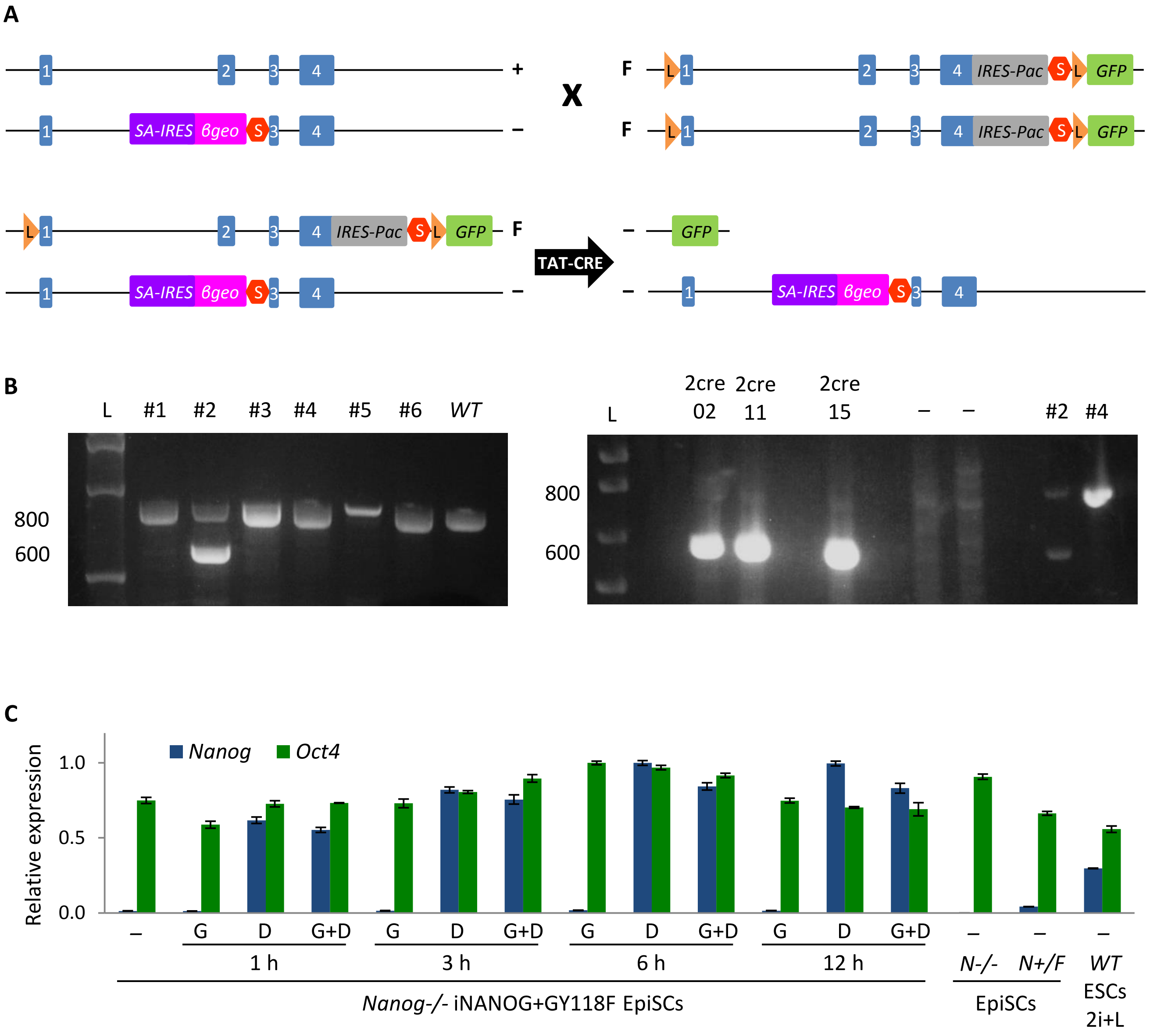
A
+
–
F
F
X
SA-IRES βgeo
IRES-Pac
GFP
TAT-CRE
B
L #1 #2 #3 #4 #5 #6 WT
800
600
L
2cre 02
2cre 11
2cre 15
–
–
#2 #4
800
600
C
Nanog
Oct4
Relative expression
1.0
0.5
0.0
– G D G+D G D G+D G D G+D G D G+D – – –
1 h
3 h
6 h
12 h
N-/- N+/F WT
Nanog-/- iNANOG+GY118F EpiSCs
EpiSCs
ESCs 2i+L

Figure S3: NANOG and pSTAT3 induce rapid and efficient reactivation of naïve genes (Related to Figure 3)

(A) Simplified schematic of the strategy used to generate *Nanog-/-* EpiSCs, not to scale. All alleles are under control of the endogenous *Nanog* promoter, and the *Nanog* 5' UTR is unchanged. We crossed *NanogF/F* [S17] and *Nanog+/-* [S18] mice and derived EpiSCs from the resulting *Nanog-/F* and *Nanog+/F* post-implantation embryos. We treated *Nanog-/F* EpiSCs with TAT-CRE, to excise *LoxP*-flanked *Nanog* exons and bring *GFP* under control of the *Nanog* promoter. This resulted in *Nanog-/-* EpiSCs with *Nanog*-promoter-driven *βgeo* and *GFP* expression. Key: floxed-*Nanog* (F); wild-type *Nanog* (+); deleted *Nanog* (-); endogenous *Nanog* exons (1,2,3,4); splice-acceptor (*SA*); internal ribosome entry site (*IRES*); geneticin resistance (*βgeo*); stop codon (*S*); *LoxP* site (*L*); puromycin resistance (*Pac*); *GFP* coding sequence without a promoter (*GFP*).

(B) Genotyping PCR was conducted with 3 primers, 1 of which is common to *F*, + and *βgeo* alleles. 1 primer recognizes both *F* and *+* alleles, to give an 800 bp product. 1 primer anneals to the *βgeo* allele, yielding a 600 bp product. L denotes a molecular weight ladder.

Left: Genotyping of embryo-derived EpiSCs revealed that #2 was *Nanog-/F*. Right: *Nanog-/-* clonal EpiSC lines (2cre02, 11, and 15) were obtained after treating #2 Nanog-*/F* EpiSCs with TAT-CRE. *Nanog-/-* ESC samples containing different knock-out alleles were included as negative controls (–), while #2 *Nanog-/F* and #4 *Nanog+/F* EpiSCs provided positive controls. *Nanog-/-* 2cre15 EpiSCs were used in all subsequent experiments.

(C) RT-qPCR analysis of *Nanog* and *Oct4* expression in *Nanog-/-* iNANOG+GY118F EpiSCs following induction with dox (D) and/or GCSF (G) in FGF2+ActivinA. Wild-type ESCs in 2i+LIF (2i+L), and *Nanog-/-* and *Nanog+/F* EpiSCs in FGF2+ActivinA conditions were included as controls. Gene expression was measured relative to *Gapdh* and normalized to the highest value. Data shown are the mean of 3 technical replicates and are from 1 of 2 representative experiments. Error bars indicate ± s.d.

Figure S4:

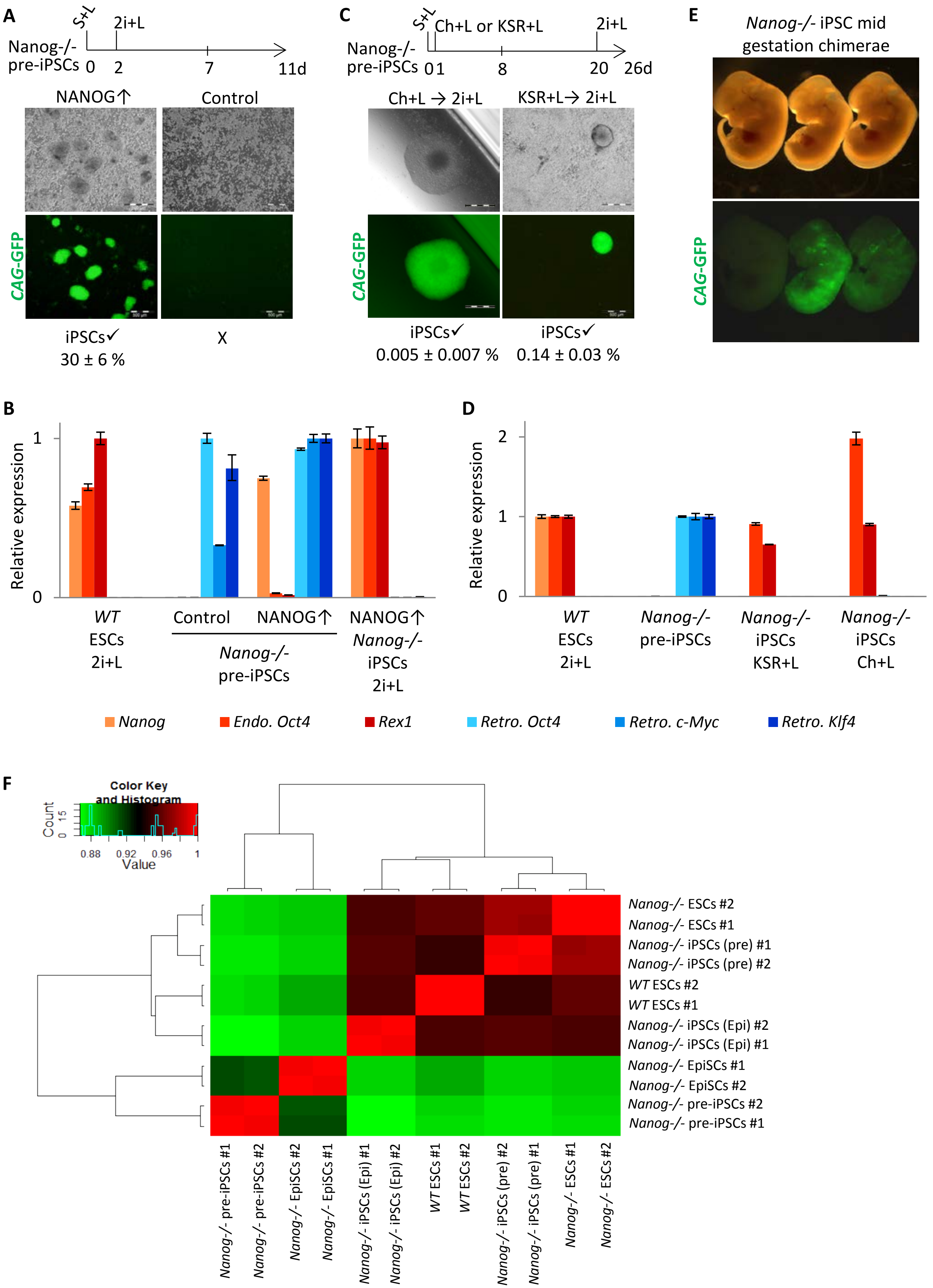
A
S+L
2i+L
Nanog-/- pre-iPSCs
0 2 7 11d
NANOG↑
Control
CAG-GFP
iPSCs✓
30 ± 6 %
X
C
S+L
Ch+L or KSR+L
2i+L
Nanog-/- pre-iPSCs
0 1 8 20 26d
Ch+L → 2i+L
KSR+L→ 2i+L
CAG-GFP
iPSCs✓
0.005 ± 0.007 %
iPSCs✓
0.14 ± 0.03 %
E
Nanog-/- iPSC mid gestation chimerae
CAG-GFP
B
Relative expression
1
0
WT ESCs 2i+L
Control
NANOG↑
Nanog-/- pre-iPSCs
NANOG↑ Nanog-/- iPSCs 2i+L
D
Relative expression
2
1
0
WT ESCs 2i+L
Nanog-/- pre-iPSCs
Nanog-/- iPSCs KSR+L
Nanog-/- iPSCs Ch+L
Nanog
Endo. Oct4
Rex1
Retro. Oct4
Retro. c-Myc
Retro. Klf4
F
Color Key and Histogram
Count
0 15
0.88 0.92 0.96 1
Value
Nanog-/- ESCs #2
Nanog-/- ESCs #1
Nanog-/- iPSCs (pre) #1
Nanog-/- iPSCs (pre) #2
WT ESCs #2
WT ESCs #1
Nanog-/- iPSCs (Epi) #2
Nanog-/- iPSCs (Epi) #1
Nanog-/- EpiSCs #1
Nanog-/- EpiSCs #2
Nanog-/- pre-iPSCs #2
Nanog-/- pre-iPSCs #1
Nanog-/- pre-iPSCs #1
Nanog-/- pre-iPSCs #2
Nanog-/- EpiSCs #2
Nanog-/- EpiSCs #1
Nanog-/- iPSCs (Epi) #1
Nanog-/- iPSCs (Epi) #2
WT ESCs #1
WT ESCs #2
Nanog-/- iPSCs (pre) #2
Nanog-/- iPSCs (pre) #1
Nanog-/- ESCs #1
Nanog-/- ESCs #2

G

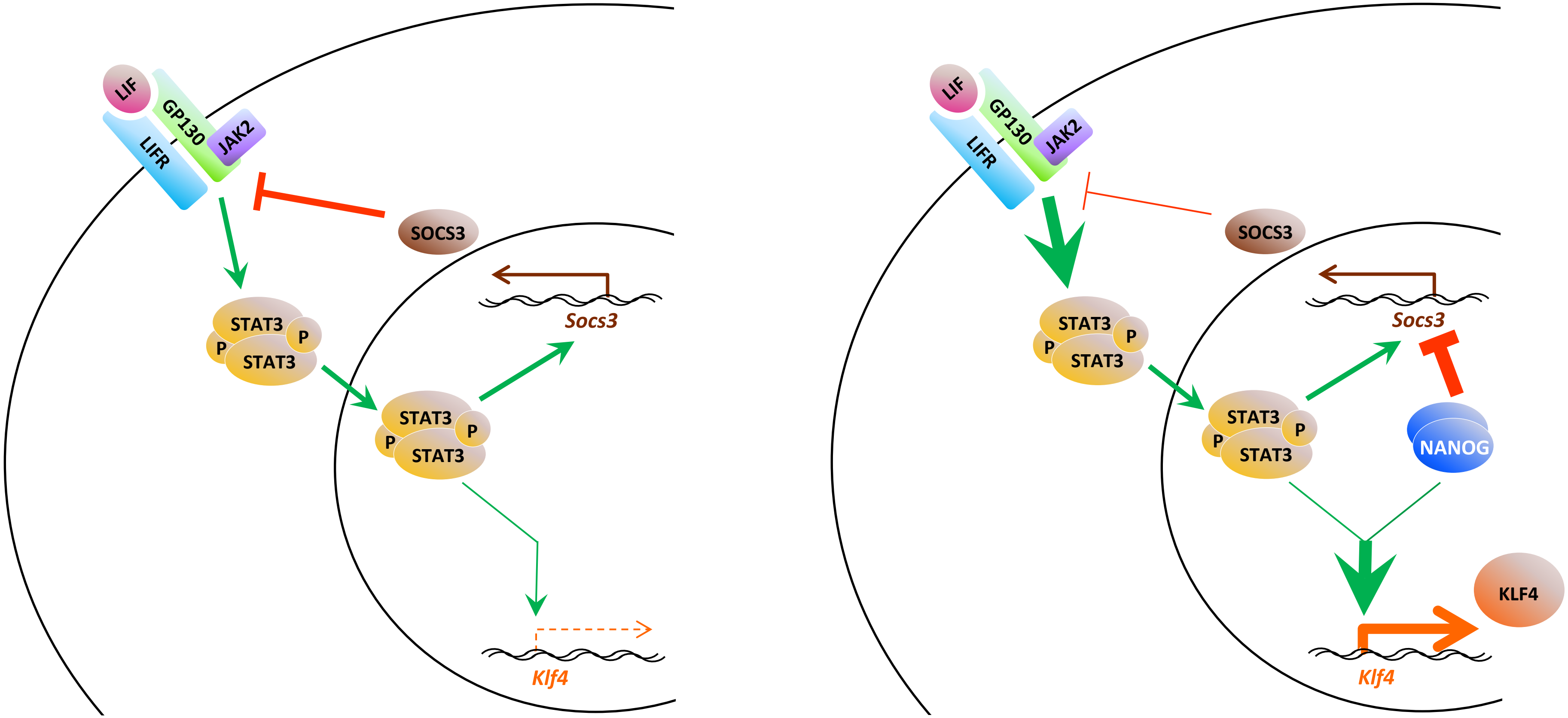
LIF
GP130
LIFR
JAK2
SOCS3
STAT3
P
STAT3
P
STAT3
P
STAT3
P
Socs3
Klf4
LIF
GP130
LIFR
JAK2
SOCS3
STAT3
P
STAT3
P
STAT3
P
STAT3
P
Socs3
NANOG
KLF4
Klf4

Figure S4: Bypass of NANOG in reprogramming (Related to Figure 4)
(A–E) Constitutively GFP+ (*CAG*-GFP) *Nanog-/-* NSCs were derived from chimerae, then converted to pre-iPSCs by retroviral transduction with *Klf4, Oct4* and *c-Myc* in serum+LIF, as previously described [S19]. Pre-iPSCs are non-pluripotent, transgene-dependent, proliferative reprogramming intermediates [S20]. NANOG is dispensable for the initial formation of pre-iPSCs, but is essential for pre-iPSCs to transit to naïve pluripotency in 2i+LIF [S19] (confirmed in A, B). Since *Nanog-/-* EpiSCs were able to reprogram in chiron+LIF but not 2i+LIF (Figure 4), we tested whether *Nanog-/-* pre-iPSCs could also reprogram in alternative conditions.
(A) *Nanog-/-* pre-iPSCs with constitutive NANOG-overexpression or control transgenes were plated in serum+LIF (S+L) and, after 2 days, medium was switched to 2i+LIF (2i+L) to prompt reprogramming. On day 7, G418 was added to select for emergent iPSCs, since *βgeo* is under control of an endogenous *Nanog* promoter. On day 11, representative phase and *CAG*-GFP images were taken and iPSC colonies were counted. The mean number of iPSC colonies is indicated as % of pre-iPSCs initially plated, ± s.d. (n=3 biological replicates).
(B) RT-qPCR analysis of gene expression in 2i+LIF-derived NANOG-rescue iPSCs, compared to parental and control pre-iPSCs in serum+LIF and wild-type ESCs in 2i+LIF. Reprogramming to naïve iPSCs was confirmed by reactivation of endogenous pluripotency genes and retroviral factor silencing. Gene expression was measured relative to *Gapdh* and normalized to the highest value. Data shown are the mean of 3 technical replicates and are from 1 of 2 representative experiments. Error bars indicate ± s.d.
(C) *Nanog-/-* pre-iPSCs were plated in serum+LIF (S+L) and, after 1 day, medium was switched to chiron+LIF (Ch+L) or KSR+LIF (KSR+L) to prompt reprogramming. On day 8, G418 was added to select for emergent iPSCs, since *βgeo* is under control of an endogenous *Nanog* promoter. On day 20, medium was switched to 2i+LIF (2i+L) to further select for naïve iPSCs. On day 26, representative phase and *CAG*-GFP images were taken and iPSC colonies were counted. The mean number of iPSC colonies is indicated as % of pre-iPSCs initially plated, ± s.d. (n=3 biological replicates). We successfully obtained *Nanog-/-* iPSCs in chiron+LIF and KSR+LIF conditions, albeit with low speed and efficiency. Like those generated from EpiSCs, the *Nanog-/-* iPSCs derived from pre-iPSCs could be maintained in 2i+LIF after naïve pluripotency establishment.
(D) RT-qPCR analysis of gene expression in chiron+LIF- and KSR+LIF-derived *Nanog-/-* iPSCs after passaging in 2i+LIF, compared to *Nanog-/-* pre-iPSCs in serum+LIF and wild-type ESCs in 2i+LIF. Reprogramming to naïve iPSCs was confirmed by reactivation of endogenous pluripotency genes and retroviral factor silencing. Gene expression was measured relative to *Gapdh* and normalized to wild-type ESC level. Data shown are the mean of 3 technical replicates and are from 1 of 2 representative experiments. Error bars indicate ± s.d.
(E) Contribution of constitutively GFP+ pre-iPSC-derived *Nanog-/-* iPSCs to mid-gestation chimerae, formally demonstrating their reacquisition of developmental pluripotency.
(F) Correlation heatmap of global gene expression between: wild-type ESCs, *Nanog-/-* ESCs, pre-iPSC-derived *Nanog-/-* iPSCs, pSTAT3+KLF4 EpiSC-derived *Nanog-/-* iPSCs all in 2i+LIF; parental *Nanog-/-* pre-iPSCs in serum+LIF; parental *Nanog-/-* EpiSCs in FGF2+ActivinA.
2 technical replicates were included for each cell line.
(G) Simplified schematic depicting the newfound role of NANOG in the modulation of LIF/STAT3 signal transduction. Left: Existing model of the LIF/STAT3 pathway (see Figure S1A for details). *Klf4* is a known target of active pSTAT3 [S5, S21, S22]. Right: We discovered that NANOG represses *Socs3* and amplifies LIF signal transduction, resulting in higher levels of active pSTAT3. Furthermore, we found that NANOG and pSTAT3 upregulate *Klf4* in a co-dependent and synergistic manner. Functionally, pSTAT3 elevation and KLF4 expression allowed us to bypass the requirement for NANOG in reprogramming.

Supplemental Experimental Procedures

Cell culture

The following established cell lines were used: wild-type E14TG2A ESCs; E14TG2A-derived *Nanog-/-* BT12 and 44cre6 ESCs [S23]; E14TG2A-derived constitutively NANOG-overexpressing RHN ESCs [S24]; *Stat3-/-* and littermate-derived *Stat3+/+* ESCs [S25]; *Nanog-/-* pre-iPSCs [S19]. ESCs and iPSCs were cultured in 2i±LIF or serum+LIF as indicated, pre-iPSCs were cultured in serum+LIF, and EpiSCs were cultured in FGF2+ActivinA. 2i medium [S25] was composed of N2B27, 3 µM CHIR99021 and 1 µM PD0325901 (Stewart lab, Dresden). N2B27 medium comprised 1:1 DMEM/F-12 and Neurobasal (Gibco), 2 mM L-glutamine (Gibco), 1x penicillin-streptomycin (Sigma), 0.1 mM 2-mercaptoethanol (Gibco), 1% B27 (Gibco) and 0.5% N2 (homemade). Serum medium contained GMEM (Sigma), 10% fetal bovine serum (Gibco), 1x non-essential amino acids (Gibco), 1 mM sodium pyruvate (Sigma), 2 mM L-glutamine (Gibco), 1x penicillin-streptomycin (Sigma) and 0.1 mM 2-mercaptoethanol (Gibco). FGF2+ActivinA medium was composed of N2B27, 12.5 ng/ml FGF2 and 20 ng/ml ActivinA (Hyvonen lab, Cambridge). During reprogramming, chiron+LIF and KSR+LIF were used when indicated. Chiron+LIF was the same as N2B27-based 2i+LIF, but without PD0325901. KSR medium contained GMEM (Sigma), 10% KnockOut Serum Replacement (Invitrogen), 1% fetal bovine serum (Gibco), 1x non-essential amino acids (Gibco), 1 mM sodium pyruvate (Sigma), 2 mM L-glutamine (Gibco), 1x penicillin-streptomycin (Sigma) and 0.1 mM 2-mercaptoethanol (Gibco). As required, media were supplemented with 20 ng/ml murine LIF (Hyvonen lab, Cambridge), 30 ng/ml human GCSF (Peprotech), or 1 µg/ml doxycycline (MP Biomedicals). For ESCs, iPSCs and pre-iPSCs, tissue-culture flasks were coated with 0.15% gelatin (Sigma) in PBS (Sigma). For EpiSCs, tissue-culture flasks were coated with 10 µg/ml fibronectin (Millipore) in PBS (Sigma). During expansion of cell lines, selection with 150 µg/ml hygromycin-B (Life Technologies), 20 µg/ml blasticidin (Gibco), 200 µg/ml G418 (Invitrogen) or 100 µg/ml zeocin (Invitrogen) was applied as appropriate. Note that G418 selects for endogenous *Nanog* promoter activity in *Nanog-/-* BT12 and 44cre6 ESCs [S23]. Colonies were dissociated with accutase (Millipore) during passaging.

Cell transfection

For transfections, 1 µg PiggyBac (PB) vector, 2 µg PBase expression vector (*pCAGPBase*) and 10 µl Lipofectamine-2000 (Invitrogen) were incubated for 20 min in 500 ml DMEM (Gibco), then applied to 500,000 cells/10 cm^2 well in 3 ml medium for 24 hours. Selection was applied to transfectants for at least 5 passages prior to use. PB-vectors stably integrate into host genome TTAA-sites in the presence of PBase transposase; *pCAGPBase* does not integrate and is lost with passaging (Cambridge Bioscience). Existing PB vectors were used to drive constitutive expression of: NANOG (*CAG-Nanog-PGK-Hph*); GY118F (*CAG-DsRed-IRES-Hph-CAG-Gy118f*, coding sequence as described in Niwa *et al*, 1998); KLF4 (*CAG-Klf4-IRES-Zeo*); ESRRB (*CAG-Esrrb-IRES-Zeo);* Control (*CAG-empty-PGK-Hph*). A selective reporter for naïve pluripotency was provided when necessary by O4GIP PB vector (*Oct4-distal-enhancer-GFP-IRES-puro*).

Derivation of EpiSCs from post-implantation embryos

EpiSCs were derived from E6.5 embryos carrying the modified alleles shown in Figure S3A. Epiblasts were manually dissected from extra-embryonic tissues and plated on fibronectin-coated plates in FGF2+ActivinA medium. After 5–7 days of culture, regions of the explant exhibiting EpiSC morphology were manually passaged to a fresh plate. Subsequent passages were performed using accutase.

Genotyping

Genomic DNA was extracted by incubation at 95°C for 20 min in 25 mM NaOH + 0.2 mM EDTA, followed by addition of an equal volume of 40 mM Tris HCl and vortexing. Genotyping PCR was conducted on the genomic DNA using Taq DNA polymerase (Qiagen) and the following thermal

cycler settings: 94°C for 5 min, 35 cycles of (94°C for 10 s, 60°C for 20 s, 72°C for 60 s), and 72°C for 3 min. An equal mix of 3 primers was used: βgeo AATGGGCTGACCGCTTCCTC; S5 ACCTCAGCCTCCAGCAGATG; A53 CAGAATGCAGACAGGTCTACAGCCCG. An 800 bp product is amplified from *Flox* and wild-type *Nanog* alleles, whereas the *βgeo* allele yields a 600 bp product.

Blastocyst injection

Chimerae were generated by standard microinjection methodology using host blastocysts of strain C57BL/6. All animal work was performed in accordance with Home Office guidelines and regulations at the University of Cambridge, UK.

Generation and characterization of doxycycline-inducible *Nanog* transgene

TetO-FLBioNanog-UbiP-rtTA3 was amplified from a previously described dox-inducible *Nanog* construct [S26]. *TetO* denotes the operator/promoter and *rtTA3* encodes the dox-controlled transactivator of the 'Tet-On' system [S27], *FLBioNanog* codes for FLAG- and BIOTIN-tagged murine NANOG, and the ubiquitous promoter *UbiP* drives constitutive expression of *rtTA3*. Amplification primers were designed to contain Gateway-compatible *attB* recombination sites: FW GGGGACAAGTTTGTACAAAAAAGCAGGCTCCGAGGTTCTAGACGAGTTTACT; RV GGGGACCACTTTGTACAAGAAAGCTGGGTCTTACCCGGGGAGCATGT. PCR was performed using Phusion Taq polymerase (New England Biolabs, recommended protocol). Successful amplification was verified on a 1% agarose gel, from which *attB1-TetO-FLBioNanog-UbiP-rtTA3-attB2* was purified using a QIAquick gel extraction kit (Qiagen). *TetO-FLBioNanog-UbiP-rtTA3* was cloned into a PiggyBac (PB) vector, to enable efficient and stable integration into the host genome. A Gateway destination PB vector containing *CAG-attR1-ccdB-attR2-IRES-Bsdr* was kindly provided by Yael Costa. To ensure that *FLBioNanog* is expressed only in the presence of dox, the constitutive *CAG* promoter was removed from the destination vector by restriction digestion and re-ligation. 150 ng of the *attB1-TetO-FLBioNanog-UbiP-rtTA3-attB2* insert was used for Gateway cloning, first into a pDonor vector using BP Clonase, then into the PB destination vector by LR Clonase (Invitrogen). Restriction digestion and DNA sequencing confirmed the production of *PB-TetO-FLBioNanog-UbiP-rtTA3-IRES-Bsdr-PB.* This resulting iNANOG construct confers constitutive blasticidin resistance (*Bsdr*) driven by *UbiP*, allowing selection of successfully transfected cells irrespective of dox presence. 1 µg/ml dox was found to be the optimal concentration for NANOG induction (data not shown). In timed induction experiments, dox was added 2 hours before GCSF, to account for the lag between transcription and translation of iNANOG (Figure S1D).

Western blotting

Dissociated cells were lysed in RIPA buffer (as described by Sigma) containing Complete-ULTRA protease-inhibitor and PhoStop phosphatase-inhibitor cocktails (Roche), and sonicated with Bioruptor200 (Diagenode) at high frequency, alternating 30 s on/off for 3 min. If necessary, lysate protein concentration was measured by BCA microplate procedure (Pierce) and adjusted prior to loading. SDS-PAGE electrophoresis was performed using NuPAGE 10% Bis-Tris gels (Invitrogen), then proteins were transferred onto a nitrocellulose membrane (Amersham) at 395 mA for 70 minutes. Membranes were stripped between pSTAT3 and total STAT3 blots (stripping buffer and protocol as described by Abcam). The following primary antibodies and dilutions were used: rabbit monoclonal against p-Y705-STAT3 (9145 1:1000) and rabbit polyclonal against total STAT3 (9132 1:1000) from Cell Signalling Technology; mouse monoclonal against OCT4 (sc-5279 1:500) from Santa Cruz Biotechnology; mouse monoclonal against α-TUBULIN (AB7291 1:5000) from Abcam; rabbit polyclonal against NANOG (A300-397A 1:5000) from Bethyl Laboratories; goat polyclonal against KLF4 (AF3158 1:1000) from R&D Systems. Detection was achieved using HRP-linked secondary antibodies against the appropriate species from GE Healthcare (anti-rabbit, anti-rat, anti-mouse) and Santa Cruz Biotechnology (anti-goat), and ECL Plus Western Blotting Detection System (GE Healthcare).

RT-qPCR

Total RNA was extracted using RNeasy kits, according to manufacturer's spin protocol, including on-column DNaseI digest (Qiagen). cDNA was produced from 1 µg RNA using SuperscriptIII VILO cDNA synthesis kit, following the recommended protocol including RNaseH treatment (Invitrogen). RT-qPCR reactions were performed in triplicate, using StepOnePlus Real Time PCR System with recommended thermocycler settings (Applied Biosystems) and TaqMan Fast Universal PCR Master Mix (Applied Biosystems). Average gene expression relative to *Gapdh* was determined using FAM-labelled TaqMan assay probe together with VIC-labelled *Gapdh* probe (Applied Biosystems).

Applied Biosystems TaqMan RT-qPCR custom assays:

**Gene**	**Forward primer (5'–3')**	**Reverse primer (5'–3')**	**Probe (FAM 5'–3' MGB)**
K4eRNA	AGGCTTTGGCTGGCTGATAA	CTGTCTCCATAGGTACTGACTTCCT	CCCAGCTCAGTAATTG
Endogenous *Oct4*	TTCCACCAGGCCCCC	GGTGAGAAGGCGAAGTCTGAAG	CCCACCTTCCCCATGGCT
Retroviral *Oct4*	TGGTACGGGAAATCACAAGTTTGTA	GGTGAGAAGGCGAAGTCTGAAG	CACCTTCCCCATGGCTG
Retroviral *Klf4*	TGGTACGGGAAATCACAAGTTTGTA	GAGCAGAGCGTCGCTGA	CCCCTTCACCATGGCTG
Retroviral *c-Myc*	TGGTACGGGAAATCACAAGTTTGTA	GGTCATAGTTCCTGTTGGTGAAGTT	CCCTTCACCATGCCCC

Applied Biosystems TaqMan RT-qPCR standard assays:

**Gene**	**Probe ID**
Gapdh	4352339E
Esrrb	Mm00442411_m1
Fgf5	Mm00438919_m1
Klf2	Mm01244979_g1
Klf4	Mm00516104_m1
Klf5	Mm00456521_m1
Nanog	Mm02384862_g1
Nr0b1	Mm00431729_m1
Nr5a2	Mm00446088_m1
Rex1	Mm03053975_g1
Socs3	Mm01249143_g1
Tfcp2l1	Mm00470119_m1

ChIP

NANOG ChIP was performed as follows: cells (10×10^6 for each sample) were fixed for 10 min in 1% formaldehyde, washed with ice-cold PBS and incubated for 10 min in lysis buffer 1 (50 mM HEPES at pH 7.5, 140 mM NaCl, 1 mM EDTA, 10% glycerol, 0.5% NP40 and 0.25% Triton X-100) and then for 10 min in lysis buffer 2 (10 mM Tris at pH 8.0, 200 mM NaCl, 1 mM EDTA and 0.5 mM EGTA). Nuclei were pelleted, resuspended in shearing buffer (1% SDS, 10 mM EDTA and 50 mM Tris at pH 8.0) and sonicated to obtain an average DNA fragment size of 500 base pairs. Lysates were diluted 1:10 in dilution buffer (50 mM Tris-HCl at pH 8.0, 167 mM NaCl, 1.1% Triton X-100 and 0.11% Na deoxycholate) and pre-cleared for 2 h at 4°C with Dynabeads Protein G magnetic beads (Life Technologies) that were pre-incubated with isotype IgG antibody. The chromatin was then incubated overnight at 4°C with 2 µg of rabbit polyclonal antibody against NANOG (Bethyl Laboratories, A300-397A) or an isotype IgG control (Santa Cruz Biotechnology, sc-2027). Lysates were then incubated for 1 h at 4°C with blocked Dynabeads magnetic beads, and the beads were washed twice in wash buffer 1 (50 mM Tris-HCl at pH 8.0, 150 mM NaCl, 1 mM EDTA, 1% Triton X-100, 0.1% SDS, 0.1% Na deoxycholate and 0.5 mM EGTA), once in wash buffer 2 (50 mM Tris-HCl at pH 8.0, 500 mM NaCl, 1 mM EDTA, 1% Triton X-100, 0.1% SDS, 0.1% Na deoxycholate and 0.5 mM EGTA), once in wash buffer 3 (50 mM Tris at pH 8.0, 250 mM LiCl, 0.5% Na deoxycholate, 0.5% NP40, 1 mM EDTA and 0.5 mM EGTA) and twice in wash buffer 4 (50 mM Tris at pH 8.0, 10 mM EDTA and 5 mM EGTA). Chromatin was eluted for 30 min at 65°C in elution buffer (1% SDS and 0.1 M $NaHCO_3$). Samples were incubated overnight at 65°C to reverse the crosslinking and purified using the QIAquick PCR Purification kit (Qiagen). Chromatin was analyzed by Fast SYBR Green RT-qPCR (Applied Biosystems). Enrichment was calculated relative to the Input sample.

ChIP primers:

**Region**	**Forward primer (5'–3')**	**Reverse primer (5'–3')**
Socs3 Proximal	GAAAAGGCTTGAGGGTCGGA	CGGGCCTGGAATGTCAAACT
Socs3 Distal	TCAGGAGTCCCTGTGCTCTAA	GGCAGACGGGTCTACTTTGAA
Negative control region	CTGGGCTTGCAGCTTAGG	AGAGACCTGGCTGAGGATGAC

Analysis of ChIP-seq datasets

The following published ChIP-seq datasets were used to examine transcription factor binding and histone modifications at genomic regions of interest in ESCs: NANOG (accession number GSE11724); pSTAT3 (GSE11431); MED12 (GSE22562); RNA PolII (GSE23943); H3K27ac (GSE24164); H3K36me3 (GSE23943); H3K4me3 (GSE23943) and H3K27me3 (GSE23943). Raw ChIP-seq datasets were downloaded from the Gene Expression Omnibus (http://www.ncbi.nlm.nih.gov/geo/), realigned to the mouse mm9 genome with Bowtie software (http://bowtie-bio.sourceforge.net), and peaks were called with MACS software (http://liulab.dfci.harvard.edu/MACS) using default parameters. 'K4-K36' domains were predicted from H3K4me3 and H3K36me3 patterns by Guttman *et al,* 2009.

Microarray

Amplification and labelling of RNA were performed using the TotalPrep-96 RNA Amplification Kit for the Illumina platform (Ambion). Subsequent hybridization, staining and scanning were performed according to the Whole Genome Gene Expression Direct Hybridization Guide on the MouseWG-6 v2.0 Expression BeadChip (Illumina). Data were loaded into the R package lumi [S28] and then divided into subsets to be analyzed. The data were transformed using Variance Stabilization [S29] and normalized using quantile normalization. Comparisons were performed in the R package limma [S30] and the results were corrected using the False Discovery Rate. Our analysis employed a 5% confidence interval.

Supplemental References

S1. Yoshida, K., Chambers, I., Nichols, J., Smith, A., Saito, M., Yasukawa, K., Shoyab, M., Taga, T., and Kishimoto, T. (1994). Maintenance of the pluripotential phenotype of embryonic stem cells through direct activation of gp130 signalling pathways. Mechanisms of development *45*, 163-171.

S2. Boeuf, H., Hauss, C., Graeve, F.D., Baran, N., and Kedinger, C. (1997). Leukemia inhibitory factor-dependent transcriptional activation in embryonic stem cells. J Cell Biol *138*, 1207-1217.

S3. Zhong, Z., Wen, Z., and Darnell, J.E., Jr. (1994). Stat3: a STAT family member activated by tyrosine phosphorylation in response to epidermal growth factor and interleukin-6. Science *264*, 95-98.

S4. Burdon, T., Smith, A., and Savatier, P. (2002). Signalling, cell cycle and pluripotency in embryonic stem cells. Trends Cell Biol *12*, 432-438.

S5. Bourillot, P.Y., Aksoy, I., Schreiber, V., Wianny, F., Schulz, H., Hummel, O., Hubner, N., and Savatier, P. (2009). Novel STAT3 target genes exert distinct roles in the inhibition of mesoderm and endoderm differentiation in cooperation with Nanog. Stem cells *27*, 1760-1771.

S6. Kershaw, N.J., Murphy, J.M., Liau, N.P., Varghese, L.N., Laktyushin, A., Whitlock, E.L., Lucet, I.S., Nicola, N.A., and Babon, J.J. (2013). SOCS3 binds specific receptor-JAK complexes to control cytokine signaling by direct kinase inhibition. Nat Struct Mol Biol *20*, 469-476.

S7. Schmitz, J., Weissenbach, M., Haan, S., Heinrich, P.C., and Schaper, F. (2000). SOCS3 exerts its inhibitory function on interleukin-6 signal transduction through the SHP2 recruitment site of gp130. The Journal of biological chemistry *275*, 12848-12856.

S8. Yoshiura, S., Ohtsuka, T., Takenaka, Y., Nagahara, H., Yoshikawa, K., and Kageyama, R. (2007). Ultradian oscillations of Stat, Smad, and Hes1 expression in response to serum. Proceedings of the National Academy of Sciences of the United States of America *104*, 11292-11297.

S9. Fukada, T., Hibi, M., Yamanaka, Y., Takahashi-Tezuka, M., Fujitani, Y., Yamaguchi, T., Nakajima, K., and Hirano, T. (1996). Two signals are necessary for cell proliferation induced by a cytokine receptor gp130: involvement of STAT3 in anti-apoptosis. Immunity *5*, 449-460.

S10. Takahashi-Tezuka, M., Yoshida, Y., Fukada, T., Ohtani, T., Yamanaka, Y., Nishida, K., Nakajima, K., Hibi, M., and Hirano, T. (1998). Gab1 acts as an adapter molecule linking the cytokine receptor gp130 to ERK mitogen-activated protein kinase. Molecular and cellular biology *18*, 4109-4117.

S11. Niwa, H., Burdon, T., Chambers, I., and Smith, A. (1998). Self-renewal of pluripotent embryonic stem cells is mediated via activation of STAT3. Genes & development *12*, 2048-2060.

S12. Burdon, T., Chambers, I., Stracey, C., Niwa, H., and Smith, A. (1999). Signaling mechanisms regulating self-renewal and differentiation of pluripotent embryonic stem cells. Cells, tissues, organs *165*, 131-143.

S13. Burdon, T., Stracey, C., Chambers, I., Nichols, J., and Smith, A. (1999). Suppression of SHP-2 and ERK signalling promotes self-renewal of mouse embryonic stem cells. Developmental biology *210*, 30-43.

S14. Marson, A., Levine, S.S., Cole, M.F., Frampton, G.M., Brambrink, T., Johnstone, S., Guenther, M.G., Johnston, W.K., Wernig, M., Newman, J., et al. (2008). Connecting microRNA genes to the core transcriptional regulatory circuitry of embryonic stem cells. Cell *134*, 521-533.

S15. Guttman, M., Amit, I., Garber, M., French, C., Lin, M.F., Feldser, D., Huarte, M., Zuk, O., Carey, B.W., Cassady, J.P., et al. (2009). Chromatin signature reveals over a thousand highly conserved large non-coding RNAs in mammals. Nature *458*, 223-227.

S16. Martello, G., Bertone, P., and Smith, A. (2013). Identification of the missing pluripotency mediator downstream of leukaemia inhibitory factor. The EMBO journal *32*, 2561-2574.

S17. Chambers, I., Colby, D., Robertson, M., Nichols, J., Lee, S., Tweedie, S., and Smith, A. (2003). Functional expression cloning of Nanog, a pluripotency sustaining factor in embryonic stem cells. Cell *113*, 643-655.

S18. Mitsui, K., Tokuzawa, Y., Itoh, H., Segawa, K., Murakami, M., Takahashi, K., Maruyama, M., Maeda, M., and Yamanaka, S. (2003). The homeoprotein Nanog is required for maintenance of pluripotency in mouse epiblast and ES cells. Cell *113*, 631-642.

S19. Silva, J., Nichols, J., Theunissen, T.W., Guo, G., van Oosten, A.L., Barrandon, O., Wray, J., Yamanaka, S., Chambers, I., and Smith, A. (2009). Nanog is the gateway to the pluripotent ground state. Cell *138*, 722-737.

S20. Silva, J., Barrandon, O., Nichols, J., Kawaguchi, J., Theunissen, T.W., and Smith, A. (2008). Promotion of reprogramming to ground state pluripotency by signal inhibition. PLoS biology *6*, e253.

S21. Niwa, H., Ogawa, K., Shimosato, D., and Adachi, K. (2009). A parallel circuit of LIF signalling pathways maintains pluripotency of mouse ES cells. Nature *460*, 118-122.

S22. van Oosten, A.L., Costa, Y., Smith, A., and Silva, J.C. (2012). JAK/STAT3 signalling is sufficient and dominant over antagonistic cues for the establishment of naive pluripotency. Nat Commun *3*, 817.

S23. Chambers, I., Silva, J., Colby, D., Nichols, J., Nijmeijer, B., Robertson, M., Vrana, J., Jones, K., Grotewold, L., and Smith, A. (2007). Nanog safeguards pluripotency and mediates germline development. Nature *450*, 1230-1234.

S24. Silva, J., Chambers, I., Pollard, S., and Smith, A. (2006). Nanog promotes transfer of pluripotency after cell fusion. Nature *441*, 997-1001.

S25. Ying, Q.L., Wray, J., Nichols, J., Batlle-Morera, L., Doble, B., Woodgett, J., Cohen, P., and Smith, A. (2008). The ground state of embryonic stem cell self-renewal. Nature *453*, 519-523.

S26. Fidalgo, M., Faiola, F., Pereira, C.F., Ding, J., Saunders, A., Gingold, J., Schaniel, C., Lemischka, I.R., Silva, J.C., and Wang, J. (2012). Zfp281 mediates Nanog autorepression through recruitment of the NuRD complex and inhibits somatic cell reprogramming. Proceedings of the National Academy of Sciences of the United States of America *109*, 16202-16207.

S27. Gossen, M., Freundlieb, S., Bender, G., Muller, G., Hillen, W., and Bujard, H. (1995). Transcriptional activation by tetracyclines in mammalian cells. Science *268*, 1766-1769.

S28. Du, P., Kibbe, W.A., and Lin, S.M. (2008). lumi: a pipeline for processing Illumina microarray. Bioinformatics *24*, 1547-1548.

S29. Lin, S.M., Du, P., Huber, W., and Kibbe, W.A. (2008). Model-based variance-stabilizing transformation for Illumina microarray data. Nucleic acids research *36*, e11.

S30. Smyth, G.K. (2004). Linear models and empirical bayes methods for assessing differential expression in microarray experiments. Stat Appl Genet Mol Biol *3*, Article3.
